# Effective delivery of STING agonist using exosomes suppresses tumor growth and enhances antitumor immunity

**DOI:** 10.1016/j.jbc.2021.100523

**Published:** 2021-03-09

**Authors:** Kathleen M. McAndrews, Sara P.Y. Che, Valerie S. LeBleu, Raghu Kalluri

**Affiliations:** 1 Department of Cancer Biology, University of Texas MD Anderson Cancer Center, Houston, Texas, USA; 2 Feinberg School of Medicine, Northwestern University, Chicago, Illinois, USA; 3 Department of Bioengineering, Rice University, Houston, Texas, USA; 4 Department of Molecular and Cellular Biology, Baylor College of Medicine, Houston, Texas, USA

**Keywords:** anticancer drug, cellular immune response, extracellular vesicles, tumor therapy, tumor microenvironment, APC, antigen-presenting cell, BMDC, bone-marrow-derived dendritic cell, CDN, cyclic dinucleotide, DC, dendritic cell, STING, Stimulator of Interferon Genes

## Abstract

The Stimulator of Interferon Genes (STING) pathway is implicated in the innate immune response and is important in both oncogenesis and cancer treatment. Specifically, activation of the cytosolic DNA sensor STING in antigen-presenting cells (APCs) induces a type I interferon response and cytokine production that facilitates antitumor immune therapy. However, use of STING agonists (STINGa) as a cancer therapeutic has been limited by unfavorable pharmacological properties and targeting inefficiency due to rapid clearance and limited uptake into the cytosol. Exosomes, a class of extracellular vesicles shed by all cells are under consideration for their use as effective carriers of drugs owing to their innate ability to be taken up by cells and their biocompatibility for optimal drug biodistribution. Therefore, we engineered exosomes to deliver the STING agonist cyclic GMP-AMP (iExo^STINGa^), to exploit their favorable pharmacokinetics and pharmacodynamics. Selective targeting of the STING pathway in APCs with iExo^STINGa^ was associated with superior potency compared with STINGa alone in suppressing B16F10 tumor growth. Moreover, iExo^STINGa^ showed superior uptake of STINGa into dendritic cells compared with STINGa alone, which led to increased accumulation of activated CD8^+^ T-cells and an antitumor immune response. Our study highlights the potential of exosomes in general, and iExo^STINGa^ specifically, in enhancing cancer therapy outcomes.

The success of immune checkpoint inhibitors in invasive cancer has renewed interest in harnessing the immune control of cancer for clinical benefit (1). Recent focus on enhancing antitumor responses, in particular for patients who remain refractory to immune checkpoint blockade, has energized the study of therapeutics that polarize the tumor microenvironment and boosting T cell response using alternative pathways. Promoting an antitumor immune microenvironment relies in part on sustained activation of T cells to eradicate cancer cells, but also on the engagement of the innate immune system (2, 3).

Dendritic cells (DCs) bridge innate and adaptative response, and DNA released from genomic unstable cancer cells elicits DCs' type I interferon signaling, activating naïve T cells and promoting antitumor responses (4, 5). A critical pathway in DCs sensing cytosolic DNA, in part serving as viral infection police, is the stimulator of interferon genes (STING) pathway. Cytosolic DNA is converted to cyclic GMP-AMP (cGAMP) by cGAS (cyclic GMP-AMP synthase). The STING protein on the endoplasmic reticulum of DCs responds to cGAMP by activating the transcription of type I interferon and cytokines, which in turn activates and primes antigen-specific CD8^+^ T cells (6, 7). In the context of tumors, STING activation can also influence the immune microenvironment by limiting the accumulation of MDSCs and Tregs and by promoting M1-macrophage polarization (4). Activation of the STING pathway promotes innate and adaptive immune cell infiltration in tumors, but also exerts antitumor effects by triggering apoptosis, inducing autophagy, and suppressing cell cycle progression of cancer cells, and by promoting vascular normalization in endothelial cells (8). Developing anticancer therapeutics by enhancing STING signaling in tumors thus may generate clinical benefit by impacting both cancer cells and the tumor microenvironment.

The utility of STING agonist (STINGa), namely cGAMP and other cyclic dinucleotides (CDNs), in the treatment of cancer is in early phase of clinical testing (7), and ongoing efforts are directed toward overcoming its unfavorable pharmacological profile and poor bioavailability (9, 10, 11, 12). Despite extensive preclinical studies indicating antitumor efficacy of STINGa, notably in synergy with other immune modulators, initial efforts in the development of pharmacological STINGa resulted in marginal benefit in patients, prompting the development of agonists with enhanced stability (13). CDNs are targeted for degradation by phosphodiesterases, in circulation and on the cell surface, severely limiting the half-life of STINGa (6, 13). In addition, cGAMP and emerging modified STINGa are hydrophilic and negatively charged, rendering them largely nonpenetrating and limiting their cellular uptake (8, 14).

The use of nanocarriers for cytosolic delivery of STINGa would presumably enhance target engagement and minimize their rapid clearance (6, 15). Exosomes are shed by all cells and are abundant in circulation and other biological fluids (16). They originate from the double invagination of the plasma membrane and released as 40–150 nm, lipid-bilayer vesicles with a surface that, in part, mimics the surface of the cells they came from (16). Toward this end, exosomes, a unique class of extracellular vesicles, are natural nanocarriers that demonstrate an efficient uptake by DCs and other APCs, with potential privilege from immune clearance (17, 18, 19, 20, 21, 22, 23, 24). Notably, T-cells-derived exosomes containing genomic and mitochondrial DNA were reported to stimulate the STING pathway in DCs (25).

We recently described the use of exosomes as a carrier for the delivery of siRNA therapeutic payload, targeting oncogenic Kras in pancreatic cancer (26, 27). We identified that exosomes were readily taken up by cancer cells, enabling superior siRNA-mediated targeting of oncogenic Kras compared with synthetic liposomes (27). CD47 on their surface limited their clearance from the circulation *via* phagocytosis, when exogenously administered to tumor bearing mice, enhancing antitumor responses (27). Exosomes likely have multiple features enabling them as natural nanocarriers for cancer therapeutics and are under active study (24). Taking advantage of our exosomes production platform (26), we tested the underlying biology and antitumor efficacy of engineered exosomes containing STINGa (iExo^STINGa^).

## Results

### Generation and validation of iExo^STINGa^

**Figure 1:**
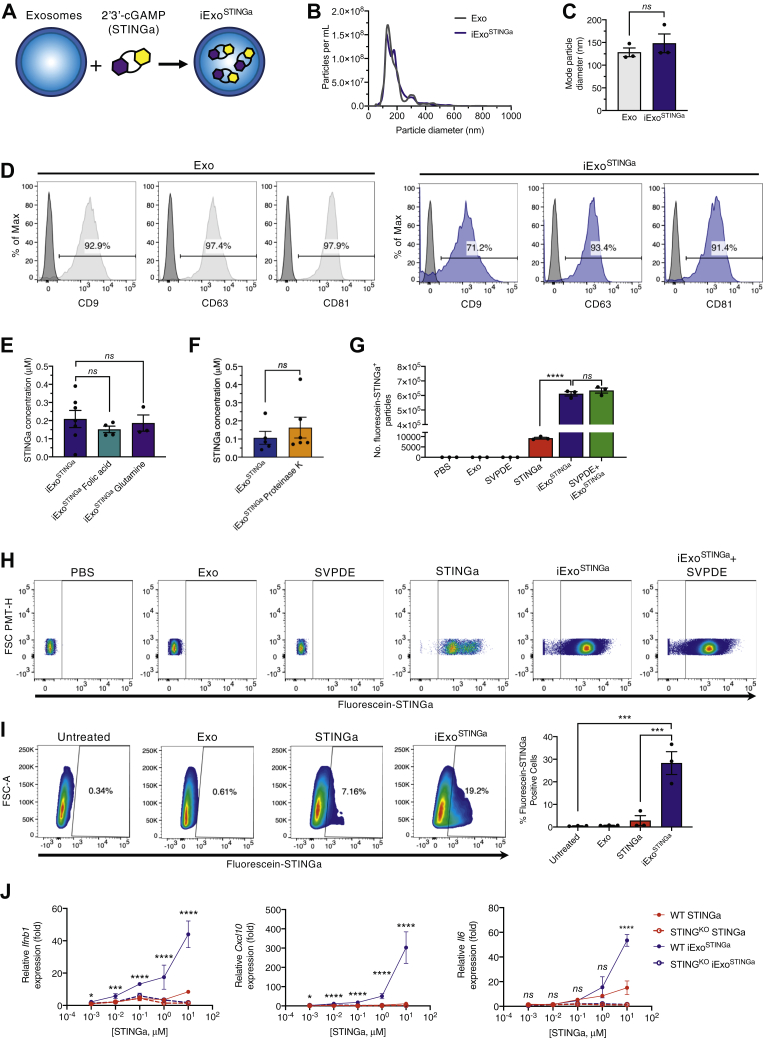
**Characterization of iExo**^**STINGa**^. *A*, schematic representation of iExo^STINGa^ generation. *B*, concentration and size distribution of purified HEK293T exosomes and iExo^STINGa^. *C*, mode particle diameter of purified HEK293T exosomes and iExo^STINGa^, n = 3 independent experiments. Mann–Whitney test performed. *D*, representative FACS histograms for CD9, CD63, and CD81 on HEK293T exosomes and iExo^STINGa^. *E*, plate reader-based quantification of fluorescein-STINGa in exosomes in the presence of 200 μM folic acid or 100 mM glutamine. n=3–7 independent experiments. One-way ANOVA with Bonferroni's multiple comparison test performed. *F*, plate reader-based quantification of fluorescein-STINGa in exosomes in the presence of Proteinase K. n = 5–6 independent experiments. Mann–Whitney test performed. *G* and *H*, representative FACS plots (*H*) and quantification of fluorescein-labeled STINGa (*G*) in the listed samples. n = 3 independent experiments. One-way ANOVA with Bonferroni's multiple comparison test performed. *H*, representative FACS plots and quantification of the percent of fluorescein-STINGa positive BMDCs in the indicated treatment groups. *I*, relative gene expression (fold change relative to untreated cells) of *Ifnb1* (*left panel*), *Cxcl10* (*center panel*), and *Il6* (*right panel*) in wildtype (WT) and STING KO BMDCs treated with varying concentrations of STINGa or iExo^STINGa^ (1 × 10^11^ HEK293T exosomes loaded with varying amounts of STINGa). One-way ANOVA with Bonferroni's multiple comparison test performed. The data are presented as the mean ± SEM. ∗ *p* < 0.05, ∗∗ *p* < 0.01, ∗∗∗ *p* < 0.001, ∗∗∗∗ *p* < 0.0001. ns, not significant.

We engineered exosomes containing the small-molecule STING agonist cyclic GMP-AMP (cGAMP, STINGa), thereafter referred to as iExo^STINGa^. Exosomes enriched from the culture supernatant of HEK293T cells were loaded with cGAMP (Fig. 1*A*, see Experimental Procedures). Nanoparticle tracking analysis revealed a similar size distribution characteristic of exosomes, and loading of exosomes with STINGa did not alter their size or concentration (Fig. 1, *B* and *C*). Both unloaded exosomes (control exosomes, Exo) and iExo^STINGa^ displayed expression of tetraspanin markers characteristic of exosomes (CD9, CD63, and CD81), as evaluated by flow cytometry analysis of surface expression (Fig. 1*D*). A standard curve employing fluorescein-labeled STINGa was developed, and it was used to estimate that approximately 200 mM STINGa is associated with exosomes (approximately 2% of STINGa, Fig. 1*E*).

In order to determine whether STINGa may bind to intraluminal STING, we evaluated the level of STING in exosomes. STING protein was not detected in HEK293T cell lysates or exosomes (Fig. S1, *A* and *B*) (28, 29, 30). Previous studies identified the folate receptor SLC19A1 as a transporter of STINGa into cells (31). Therefore, we evaluated SLC19A1 protein levels in HEK293T cells and exosomes, and SLC19A1 was not detected in exosomes (Fig. S1, *C* and *D*). In agreement with these findings, incubation with folic acid, which competes for folic acid receptors, did not alter the amount of STINGa associated with the exosomes (Fig. 1*E*). The glutamine/glutamate transporter, SLC38A2, another potential transporter of STINGa was assessed, and it was identified in HEK293T cells but not the exosomes (Fig. S1, *E* and *F*). Incubation with glutamine to compete with STINGa for glutamine/glutamate transporters did not alter STINGa content in the exosomes (Fig. 1*E*). Treatment of exosomes with proteinase K to cleave all surface proteins and ectodomains of transmembrane proteins (Fig. S1*G*) that could be potentially involved in STINGa transport did not significantly alter STINGa present in the exosomes (Fig. 1*F*). Together, these data suggest that STINGa may enter exosomes in a passive manner without involvement in exosomal surface proteins.

To evaluate the association of fluorescein-labeled STINGa with exosomes, small-particle flow cytometry was performed. Fluorescein-STINGa^+^ particles were specifically detected in iExo^STINGa^ when compared with Exo (Fig. 1, *G* and *H*), and treatment with snake venom phosphodiesterase (SVPDE) to cleave extraluminal STINGa did not reduce the number of STINGa^+^ particles (Fig. 1, *G* and *H*), suggesting that STINGa is found predominantly within the lumen of exosomes.

Uptake of iExo^STINGa^ in DCs was evaluated using bone-marrow-derived dendritic cells (BMDCs) from wildtype and *Sting1* knock out (STING^KO^) mice. The uptake of fluorescently labeled STINGa in BMDCs was superior when using iExo^STINGa^ compared with STINGa (Fig. 1*I*). BMDCs treated with iExo^STINGa^ also showed increased expression of *Ifnb1*, *Cxcl10*, and *Il6* when compared with cells treated with STINGa (Fig. 1*J*). The transcriptional upregulation of these genes, supporting STING pathway activation, was not observed in STING^KO^ BMDCs, indicating a specific target engagement (Fig. 1*J*).

### STING activation with exosomes shows antitumor response

**Figure 2:**
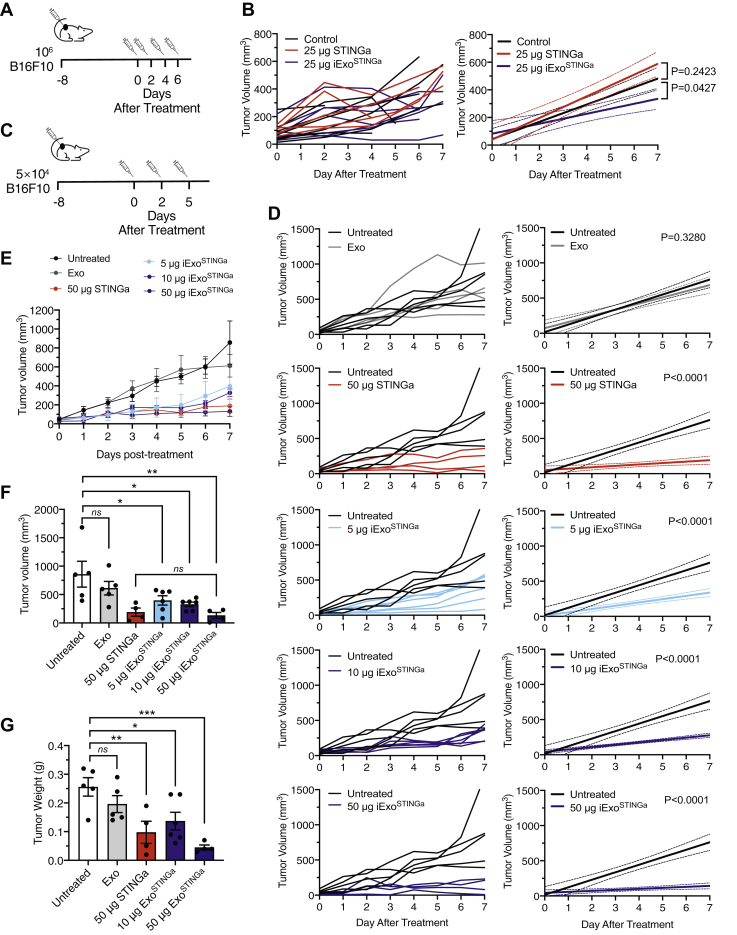
**Antitumor activity of iExo**^**STINGa**^. *A*, schematic representation of experiment with 25 μg iExo^STINGa^ treatment. *B*, tumor volume over time of individual mice in the indicated groups (*left panel*). Tumor volume over time in select groups and linear regression analysis testing for significant differences in slope (*right panel*). *C*, schematic representation of experiment with 5, 10, or 50 μg iExo^STINGa^ treatment. *D–G*, tumor volume over time of individual mice in the indicated groups (*D*, *left panels*), tumor volume over time in select groups, and linear regression analysis testing for significant differences in slope (*D*, *right panels*), tumor growth kinetics (*E*), endpoint tumor volumes (*F*), and endpoint tumor weights (*G*) in the indicated groups. *F* and *G*, one-way ANOVA with Bonferroni's multiple comparison test performed. The data are presented as the mean ± SEM. ∗ *p* < 0.05, ∗∗ *p* < 0.01, ∗∗∗ *p* < 0.001. ns, not significant.

The impact of iExo^STINGa^ on tumor growth was tested using immunocompetent mice implanted with subcutaneous B16F10 tumors. Large inoculum of B16F10 generated tumors with rapid growth kinetics (Fig. 2*A*). Compared with control mice, 25 μg STINGa failed to suppress growth, while 25 μg iExo^STINGa^ reduced tumor growth (Fig. 2*B*, Fig. S2, *A* and *B*). A low dose of 0.5 μg iExo^STINGa^ failed to impact tumor growth (Fig. S2, *A* and *B*). Experiments were subsequently performed with smaller B16F10 inoculum (STINGa responsive condition), enabling the comparison of STINGa with iExo^STINGa^ (Fig. 2*C*). When mice presented with 50 mm^3^ tumors, treatment was initiated, and experimental groups received three intratumoral injections of the indicated concentration of iExo^STINGa^, STINGa, or just exosomes (Exo) (Fig. 2*A*). Treatment with 5 μg iExo^STINGa^, 10 μg iExo^STINGa^, 50 μg iExo^STINGa^, or 50 μg STINGa showed a dose-depending antitumor response compared with Exo and untreated controls (Fig. 2, *D* and *E*). At experimental endpoint, tumor volume and tumor weight were significantly reduced in experimental groups receiving 10 μg iExo^STINGa^, 50 μg iExo^STINGa^, or 50 μg STINGa, when compared with Exo and untreated controls (Fig. 2, *F* and *G*).

### Induction of T-cell activation with iExo^STINGa^ enables systemic antitumor response

**Figure 3:**
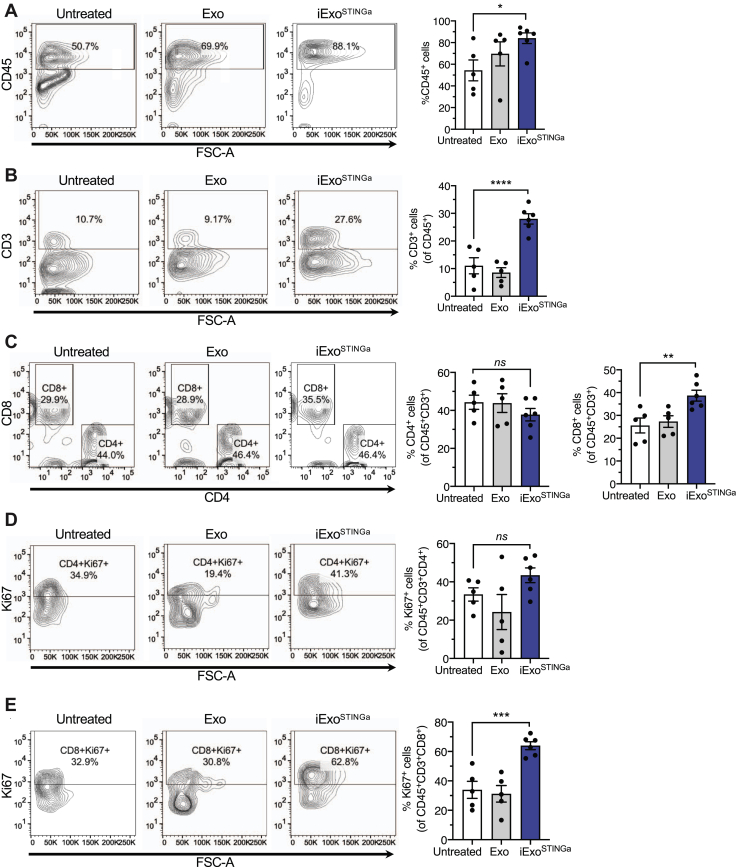
**T cells infiltration in iExo**^**STINGa**^**-treated tumors.***A–D*, quantitative analyses of flow cytometry measurement of CD45^+^ (*A*), CD3^+^ (*B*), CD4^+^, and CD8^+^ (*C*) cells in the tumors of the indicated groups. *A*, Kruskall–Wallis with Dunn's multiple comparison test performed; *B* and *C*, one-way ANOVA with Bonferroni's multiple comparison test performed. *D* and *E*, quantitative analyses of flow cytometry measurement of CD4^+^Ki67^+^ (*D*) and CD8^+^Ki67^+^ (*E*) cells in the tumors of the indicated groups. *D* and *E*, one-way ANOVA with Bonferroni's multiple comparison test performed. The data are presented as the mean ± SEM. ∗ *p* < 0.05, ∗∗ *p* < 0.01, ∗∗∗ *p* < 0.001, ∗∗∗∗ *p* < 0.0001. ns, not significant.

**Figure 4:**
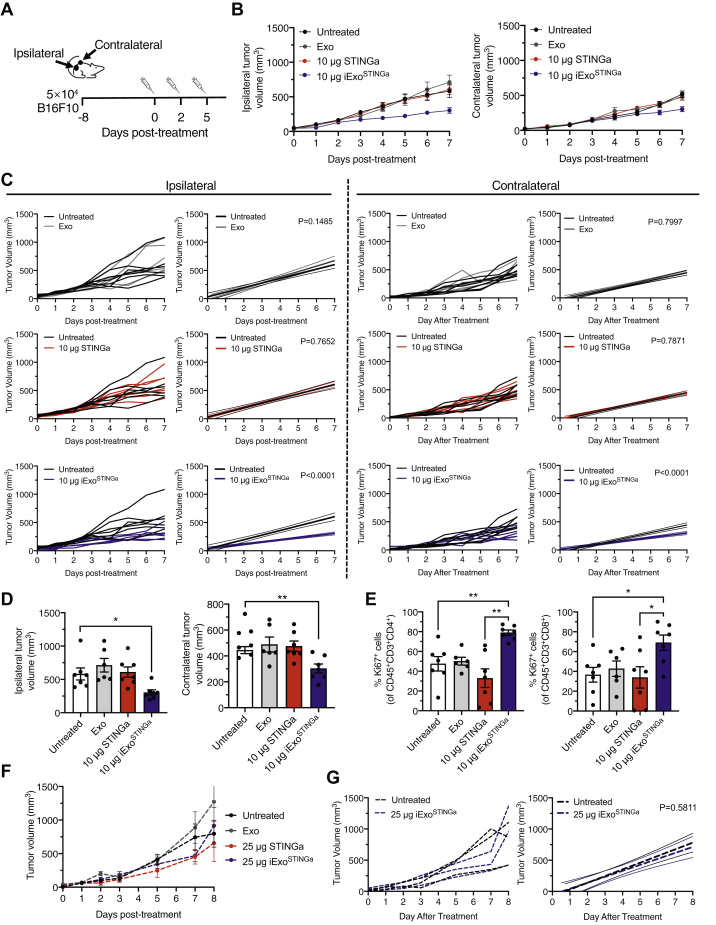
**Systemic antitumor effect of iExo**^**STINGa**^. *A*, schematic representation of experiment. *B*, volume of the ipsilateral (*left panel*) and contralateral (*right panel*) tumors over time in the indicated groups. *C*, ipsilateral (*left panels*) and contralateral (*right panels*) tumor volume over time in select groups and linear regression analysis testing for significant differences in slope. *D*, endpoint tumor volumes of ipsilateral (*left panel*) and contralateral (*right panel*) tumors. One-way ANOVA with Dunnett's multiple comparison test performed. *E*, quantitative analyses of flow cytometry measurement of CD4^+^Ki67^+^ (*left panel*) and CD8^+^Ki67^+^ (*right panel*) cells in the ipsilateral tumors of the indicated groups. For *left panel*, Brown–Forsythe and Welch ANOVA with Dunnett's T3 multiple comparison test performed. For *right panel*, one-way ANOVA with Bonferonni's multiple comparison test performed. *F*, tumor volumes over time in the indicated groups. *G*, contralateral tumor volume over time in select groups (*left panel*) and linear regression analysis testing for significant differences in slope (*right panel*). The data are presented as the mean ± SEM. ∗ *p* < 0.05, ∗∗ *p* < 0.01.

To ascertain the underlying mechanism associated with the antitumor activity of iExo^STINGa^, the immune microenvironment of iExo^STINGa^-treated tumors was evaluated. A significant increase in CD45^+^, CD3^+^, and CD8^+^ immune cells was observed in iExo^STINGa^-treated tumors compared with untreated, Exo-treated control tumors (Fig. 3, *A*–*C*). CD4^+^ and proliferating (Ki67^+^) CD4^+^ T cells were unchanged, but proliferating CD8^+^ T cells were increased in iExo^STINGa^-treated tumors (Fig. 3, *C*–*E*). Indication of a systemic influence on immune control of tumor with iExo^STINGa^ therapy was evidenced with a reduction in contralateral tumor growth when ipsilateral tumors are treated. Mice were implanted with tumors on both flanks, and ipsilateral tumors were treated as described above (and shown in Fig. 4*A*). Tumor growth suppression in the ipsilateral (receiving intratumoral treatment) tumor was significant in iExo^STINGa^-treated mice when compared with control and superior to STINGa-treated mice (Fig. 4, *B*–*D*). A significant tumor growth suppression was also evident in the contralateral tumor in iExo^STINGa^-treated mice when compared with control mice, whereas STINGa did not significantly suppress contralateral tumor growth (Fig. 4, *B*–*D*). The suppression of ipsilateral tumors with iExo^STINGa^ was associated with a significant increase in proliferating CD4^+^ and CD8^+^ T cells when compared with STINGa-treated tumors and control tumors (Fig. 4*E*, Fig. S3, *A* and *B*).

To confirm the *in vivo* specificity of iExo^STINGa^ on the STING pathway, we treated B16F10 tumor-bearing STING^KO^ mice with 25 μg iExo^STINGa^ or 25 μg STINGa. Tumor growth was not significantly different when B16F10 tumors are implanted in STING^KO^ mice compared with wildtype mice (Fig. S3*C*). Tumor volumes measurements indicated that 25 μg iExo^STINGa^ or 25 μg STINGa failed to suppress tumor growth on the STING^KO^ background (Fig. 4, *F* and *G*, Fig. S3*D*). These results collectively support that the antitumor response exerted by iExo^STINGa^ is realized by engaging its specific target (STING pathway). Finally, we tested the stability of by iExo^STINGa^ in stimulating BMDCs. Storage of iExo^STINGa^ at –20 °C for 2 weeks or –80 °C for 1 month before thaw and use did not significantly impair increased expression of *Ifnb1*, *Cxcl10*, and *Il6*, when compared with freshly prepared iExo^STINGa^ (Fig. S3, *E*–*G*).

## Discussion

We report on the efficacy of exosomes as carrier of STINGa for antitumor therapy. STINGa was predominantly found intraluminally in the iExo^STINGa^ and was resistant to SVPDE degradation. The entry of STINGa into exosomes appears to be due to potential passive diffusion through the lipid bilayer. iExo^STINGa^ showed a markedly superior activation of the STINGa pathway and activation of DCs, when compared with STINGa. This is likely as a result of enhanced uptake or retention of iExo^STINGa^ in DCs compared with using STINGa by itself. Our results indicate iExo^STINGa^ showed approximately tenfold increase in uptake by DCs compared with free STINGa. These results support the previously reported efficacy of exosomes in delivering a therapeutic payload into the cytosol of treated cells (16, 23, 27, 32). Liposomes, polymer nanoparticles, and hydrogels have been studied to enhance STINGa cytosolic delivery and stability, with mixed results (6). Nonetheless, these efforts support a potential use of a carrier for STINGa-based therapy to overcome the pharmacological limitations of STINGa. The added benefit of iExo^STINGa^ compared with synthetic carriers may lie in the enhanced stability of exogenously administered exosomes, with lipid and protein compositions that do not elicit phagocytic clearance. Interestingly, the packaging of cGAMP in viral particles, concurrently studied with exosomes, indicated superior transfer of cGAMP in using viral particles (33). This study used exosomes from transfected cells and differs from our approach of engineering exosomes containing cGAMP, but nonetheless supports that exosomes' cargo includes cGAMP. In a related study, exosomes from irradiated cancer cells were shown to transfer dsDNA and stimulate the STING pathway in DCs (34), supporting the exosomes cargo's capacity for STING pathway activation.

Our data also indicate a superior antitumor effect of iExo^STINGa^ treatment compared with STINGa, and the reduced tumor growth with iExo^STINGa^ was associated with an influx of proliferating CD8^+^ T cells, in agreement with previous studies reporting antitumor response with STING pathway activation (4, 35). Despite being administered intratumorally, iExo^STINGa^ treatment showed an antitumor effect on contralateral, noninjected tumors. This was not observed when STINGa was used by itself. The activation of the STING pathway in the iExo^STINGa^-injected tumors generates abscopal effect that impact non-iExo^STINGa^-injected tumors in the same mice. Such response with STING pathway activation has been previously reported in the context of radiation and immune checkpoint blockade (13, 36). We speculate that such systemic changes include activation of adaptive immunity reaching other tumor sites. Exosomes are now being implicated in adaptive and immune response regulation. Though we did not observe control exosomes (deprived of STINGa cargo) eliciting measurable immune responses at the dosage reported here, it is possible that added benefit could be realized with iExo^STINGa^ generated from a cell source with potential for T-cell activation (37, 38). In this study, the robust antitumor response using iExo^STINGa^ as a single agent with systemic effect on secondary tumors supports the potential of iExo^STINGa^ in clinical use *via* an established GMP-exosomes production platform (26).

## Experimental procedures

### Cell culture

HEK293T (fetal human epithelial kidney cells, HEK 293T/17 obtained from ATCC, CRL-11269, and STR validated) and B16F10 (obtained from MD Anderson Cell Repository and CellCheck and IMPACT tested by IDEXX) were cultured in DMEM (Corning) supplemented with 10% FBS (Gemini) and 1% penicillin-streptomycin (Corning) at 37 °C/5% CO_2_, and tested negative for *mycoplasma*. THP-1 (from ATCC, TIB-202) was cultured in RPMI (Corning) with 10% FBS and 1% penicillin-streptomycin, STR validated, and tested negative for *mycoplasma*. *Sting1* knockout mice (STING^KO^) were obtained from Jackson Laboratory (Stock No: 025805), and BMDCs were generated by culturing bone marrow cells flushed from the femurs of C57BL/6J (Jackson Laboratory) and STING^KO^ mice. The unfractionated marrow was expanded for 8 days in DC medium, composed of DMEM supplemented with 10% FBS, penicillin-streptomycin, and 20 ng/ml mouse GM-CSF and refreshed every 48 h.

### Isolation and purification of exosomes

HEK293T cells were grown in T225 flasks with DMEM supplemented with 10% FBS and 1% penicillin-streptomycin at 37 °C/5% CO_2_ for 2–3 days until they reached a confluency of 60–70%. The cells were washed twice with PBS (Corning Catalog # 21-040-CV) and fed serum-free medium for 48 h. The media was collected and centrifuged at 800*g* for 5 min, followed by centrifugation at 2000*g* for 10 min. The conditioned medium was filtered using a 0.2 μm filter flask (Thermo Fisher) and ultracentrifuged at 100,000*g* for 3 h at 4 °C in a SW 32 Ti rotor (Beckman Coulter). The exosomes were resuspended in PBS, and their concentration and size distribution were evaluated using NanoSight LM10 before storage at –80 °C.

### Generation of iExo^STINGa^

For *in vitro* studies, 10^11^ HEK293T exosomes were incubated with 10 μM STINGa (2′3′-cGAMP, Invivogen, tlrl-nacga23) with rotation, at room temperature, for 16 h. Samples were then washed two times with PBS with a 30 kDa Amicon Ultra filter (EMD Millipore) according to manufacturer's instructions. In some experiments, fluorescein-labeled STINGa (fluorescein-labeled 2′3′-cGAMP, BioLog, C 178-001) was incubated with 10^11^ HEK293T exosomes at room temperature for 16 h. PBS (Corning Catalog # 21-040-CV) was used as diluent for both *in vivo* and *in vitro* studies employing STINGa or iExo^STINGa^. Characterization of STINGa on the surface or within exosomes was performed using snake venom phosphodiesterase (SVPDE, Abnova, P5263). Samples were incubated with 1 mU SVPDE for 30 min at 37 °C in 40 mM Tris and 10 mM MgCl_2_, pH 7.8, followed by enzyme inactivation at 75 °C for 10 min. For *in vivo* studies, 10^11^ HEK293T exosomes were incubated with the indicated amounts of STINGa (0.5, 5, 10, 25, 50 μg STINGa) with rotation, at room temperature, for 16 h. Samples were then washed two times with PBS with a 30 kDa Amicon Ultra filter according to manufacturer's instructions.

### Flow cytometry analysis of exosomes

Exosomes (5 × 10^9^ quantified by NanoSight analysis) were incubated with 4 μm aldehyde/sulfate latex beads (Invitrogen, A37304) for 2 h at room temperature. This suspension was diluted to 1 ml with PBS, and the reaction was stopped with incubation with 100 mM glycine for 30 min. Exosome-bound beads were blocked with 10% BSA for 1 h and stained with 50 μg/ml mouse IgG1κ isotype control (BD Bioscience, 555746), CD9 (Sigma-Aldrich, SAB4700092), CD63 (BD Bioscience, 556019), and CD81 (BD Bioscience, 555675) in 20 μl of 2% BSA in PBS, and mixed at room temperature for 1 h. Secondary anti-mouse antibodies with Alexa Fluor 488 (Life Technologies, A21202) or Alexa Fluor 647 (Life Technologies, A31571) were added, and mixed at room temperature for 1 h. Detection of CD9, CD63, and CD81 on beads was analyzed using a BD LSR Fortessa X-20. Positive signal for CD9, CD63, and CD81 was determined based on the signal in isotype control samples using FlowJo (BD Bioscience).

In order to analyze fluorescein-STINGa loaded exosomes, samples were incubated with total exosome isolation reagent from cell culture media (Invitrogen, 4478359) overnight at 4 °C, then centrifuged at 15,000 rpm for 1 h. The supernatant was collected and the pellet resuspended in PBS for analysis. Samples were analyzed with an acquisition time of 2 min using a BD LSR Fortessa X-20 cell analyzer equipped with a FSC PMT small-particle detector. Size gating of exosomes was performed based on the SSC-H *versus* FSC PMT-H distribution of 100 nm FITC beads. Positive fluorescein signal was established based on exosome samples without STINGa using FlowJo.

### Quantification of STINGa loading

For quantification of STINGa loaded in exosomes, 10^11^ exosomes were treated with 5 mg/ml proteinase K (Qiagen) for 30 min at 37 °C followed by 20 min at 60 °C. For competition assays, 200 μM of folic acid (Sigma Aldrich) or 100 mM of glutamine (Corning) was added to the exosomes. Fluorescein-STINGa (10 μM) was incubated with exosomes at room temperature for 16 h. Samples were incubated with total exosome isolation reagent from cell culture media overnight at 4 °C, then centrifuged at 15,000*g* for 1 h. The pellet was resuspended in 100 μl of PBS and analyzed on a plate reader (Omega) with an excitation of 485 nm and emission of 520 nm. STINGa concentration in exosomes was calculated based on a standard curve of Fluorescein-STINGa. For verification of proteinase K activity, samples were incubated with 4 μm aldehyde/sulfate latex beads and analyzed by FACS as described above.

### Western blot

Cell lysate was collected in 8 M urea with 2.5% SDS. Protein concentration was measured with Qubit Protein Assay (Thermo Fisher), with 30 μg of cell lysate and 15 μg of exosome protein loaded. Protein was denatured in 4× NuPAGE LDS sample buffer with 62.5 mM DTT for 10 min at 70 °C. Membranes were blocked with 5% milk in TBS with 0.1% Tween (TBST) and incubated with rabbit anti-SLC19A1 (Boster Biological Technology PB9504, 1:1000), rabbit anti-SLC38A2 (Sigma Aldrich SAB4502246, 1:1000), mouse anti-CD81 (Santa Cruz Biotechnology sc-166029, 1:1000), or rabbit anti-vinculin (Abcam ab129002, 1:1000) for 1 h at room temperature or overnight at 4 °C. Membranes were incubated in anti-rabbit HRP (Abcam ab16284, 1:1000) or anti-mouse HRP (R&D Systems HAF007, 1:1000) for 1 h at room temperature and developed using West-Q Pico ECL solution (GenDEPOT).

### *In vitro* evaluation of STINGa and iExo^STINGa^ activity and cellular uptake in BMDCs

BMDCs were seeded at 200,000 cells per well in 24-well plates and treated with iExo^STINGa^ (10^11^ exosomes), STINGa, HEK293T exosomes (Exo), or PBS for 24 h, respectively, with indicated concentrations of STINGa. RNA was isolated with RNeasy kit (Qiagen), cDNA synthesis performed with High Capacity Reverse Transcription Kit (Applied Biosystems), and qRT-PCR performed with Power SYBR Green PCR Master Mix (Applied Biosystems). Gene expression levels were normalized to the levels of housekeeping genes *Gapdh* or *Actb*. Relative gene expression is presented as fold change (2^−ΔΔCt^) with untreated cells (control group) set to a value of 1. Primer sequences are as follows: *Actb* Forward: 5′-GGCTGTATTCCCCTCCATCG-3′; *Actb* Reverse: 5′-CCAGTTGGTAACAATGCCATGT-3′; *Gapdh* Forward: 5′-AGGTCGGTGTGAACGGATTTG-3′; *Gapdh* Reverse: 5′-TGTAGACCATGTAGTTGAGGTCA-3′; *Ifnb1* Forward: 5′-GGAAAGATTGACGTGGGAGAT-3; *Ifnb1* Reverse: 5′-CAGGCGTAGCTGTTGTACTT-3; *Cxcl10* Forward: 5′-GCTGCAACTGCATCCATATC-3; *Cxcl10* Reverse: 5′-CGTGGCAATGATCTCAACAC-3; *Il6* Forward: 5′- CTTCCATCCAGTTGCCTTCT-3; *Il-6* Reverse: 5′-CTCCGACTTGTGAAGTGGTATAG-3′. Statistical analysis of qRT-PCR data was performed based on ΔC_t_ values. In order to quantify STINGa uptake, BMDCs were seeded at 200,000 cells per well in 24-well plates and treated with fluorescein-labeled free STINGa or iExo^STINGa^ for 24 h and quantified by flow cytometry analysis.

### Treatment of B16F10 subcutaneous tumors

B16F10 cells (10^6^ or 5 × 10^4^ cells in 100 μl of PBS, as specific in the figure) were injected subcutaneously into the flank of 8–12-week-old female C57BL/6J mice purchased from the Jackson Laboratory. Tumor volumes were measured every day using digital calipers and calculated using the equation length × width^2^ × 0.52. Unless otherwise stated, when the tumors reached a size of approximately 50 mm^3^, the mice were randomly assigned to the distinct treatment groups: untreated, STINGa (25 or 50 μg STINGa, 20 μl), Exo (10^11^ exosomes, 20 μl), or iExo^STINGa^ (0.5, 5, 10, 25 or 50 μg STINGa in 10^11^ exosomes, 20 μl). Each treatment was administered intratumorally every 48 to 72 h for a total of 3–4 consecutive treatments (as detailed in the figures). Mice were euthanized when a tumor burden endpoint of less than 2000 mm^3^ was reached. In specified experiments (Fig. 4), 8–12-week-old female C57BL/6J mice (Jackson Laboratory) were injected subcutaneously with B16F10 cells (5 × 10^4^ in 100 μl of PBS) on each of its flank. When the larger of the two tumors reached a volume of 50 mm^3^, intratumoral treatment was initiated. The treated tumor was referred to as the ipsilateral tumor, and the untreated tumor on the opposite flank was referred to as the contralateral tumor. Treatment groups include untreated mice and mice treated with STINGa (10 μg STINGa, 20 μl), Exo (10^11^ exosomes, 20 μl), or iExo^STINGa^ (10 μg STINGa in 10^11^ exosomes, 20 μl). Each treatment was administered intratumorally every 48–72 h for a total of three consecutive treatments. Mice were euthanized when a tumor burden endpoint of less than 2000 mm^3^ was reached. All mice were housed under standard housing conditions at MD Anderson Cancer Center (MDACC) animal facilities, and all animal procedures were reviewed and approved by the MDACC Institutional Animal Care and Use Committee.

### Flow cytometric analysis of B16F10 tumors

Tumors were dissociated with gentleMACS (Miltenyi Biotec) and digested in a solution of DNase I (Roche, 10104159001) and Liberase TL (Roche, 5401020001) in RPMI 1640 media for 30 min at 37 °C. Tumors were strained through a 40 μm cell strainer. Cells were diluted in PBS containing 2% FBS and 100 μl of cell suspension was used for surface and live/dead staining: CD45 (Pacific Blue, BioLegend, 103126, 1:100), CD3 (PE-Cy7, Invitrogen, 25-0031-82, 1:200), CD4 (BV605, BioLegend, 100548, 1:200), CD8 (BV650, BioLegend, 100742, 1:200), Live/dead eFluor 780 (eBioscience, 65-0865-14, 1:1000), and Fc block (aCD16/CD32, Invitrogen 40-0161-M001, 1:100). Cell suspensions were then fixed and permeabilized with fixation/permeabilization buffer (eBioscience) for intracellular staining of Ki67 (Alexa Fluor 488, BD Bioscience, 558616, 1:100). All flow cytometry data were analyzed using FlowJo software.

### Statistical analyses

Statistical analyses were performed in using GraphPad Prism (GraphPad Software) and the respective statistical tests used are indicated in the figure legends. Normal distribution of data was evaluated using Shapiro–Wilk and Kolmogorov–Smirnov tests. An unpaired, two-tailed *t*-test was performed for comparison of two normally distributed groups, or one-way ANOVA with Bonferroni's multiple comparison test for three or more normally distributed groups. For two groups that were not normally distributed, a Mann–Whitney test was performed. An unpaired *t*-test with Welch's correction was used for two groups that had significantly different standard deviations. Kruskall–Wallis test with Dunn's multiple comparison test was used to compare three or more groups that were not normally distributed. For comparisons that had significant differences in the standard deviations across three or more groups, Brown–Forsythe and Welch ANOVA with Dunnett's T3 multiple comparison test was performed. Linear regression analyses of averaged tumor volumes over time per experimental group were used to determine if the slopes between two groups were different. The *p* value reported on the linear regressions informs on the significant difference between the slopes. A *p* value <0.05 was considered statistically significant. Error bars represented standard error of the mean (S.E.M.).

## Data availability

The source data for the work presented in this article is included as a supporting information file.

## Supporting information

This article contains supporting information.

## Conflict of interest

V. S. L. is a paid consultant for Codiak Biosciences. MD Anderson Cancer Center and R. K. hold patents in the area of exosome biology (unrelated to the topic of this publication) and are licensed to Codiak Biosciences Inc. MD Anderson Cancer Center and R. K. are stock equity holders in Codiak Biosciences Inc. R. K. is a consultant and a scientific advisor of Codiak Biosciences Inc.

## References

[1] Sharma P., Allison J.P. (2020). Dissecting the mechanisms of immune checkpoint therapy. Nat. Rev. Immunol..

[2] Iwasaki A., Medzhitov R. (2015). Control of adaptive immunity by the innate immune system. Nat. Immunol..

[3] Gonzalez H., Hagerling C., Werb Z. (2018). Roles of the immune system in cancer: From tumor initiation to metastatic progression. Genes Dev..

[4] Hoong B.Y.D., Gan Y.H., Liu H., Chen E.S. (2020). cGAS-STING pathway in oncogenesis and cancer therapeutics. Oncotarget.

[5] Vatner R.E., Janssen E.M. (2019). STING, DCs and the link between innate and adaptive tumor immunity. Mol. Immunol..

[6] Wu J.J., Zhao L., Hu H.G., Li W.H., Li Y.M. (2020). Agonists and inhibitors of the STING pathway: Potential agents for immunotherapy. Med. Res. Rev..

[7] Motedayen Aval L., Pease J.E., Sharma R., Pinato D.J. (2020). Challenges and opportunities in the clinical development of STING agonists for cancer immunotherapy. J. Clin. Med..

[8] Zhu Y., An X., Zhang X., Qiao Y., Zheng T., Li X. (2019). STING: A master regulator in the cancer-immunity cycle. Mol. Cancer.

[9] Wilson D.R., Sen R., Sunshine J.C., Pardoll D.M., Green J.J., Kim Y.J. (2018). Biodegradable STING agonist nanoparticles for enhanced cancer immunotherapy. Nanomedicine.

[10] Miyabe H., Hyodo M., Nakamura T., Sato Y., Hayakawa Y., Harashima H. (2014). A new adjuvant delivery system ‘cyclic di-GMP/YSK05 liposome’ for cancer immunotherapy. J. Control Release.

[11] Watkins-Schulz R., Tiet P., Gallovic M.D., Junkins R.D., Batty C., Bachelder E.M., Ainslie K.M., Ting J.P.Y. (2019). A microparticle platform for STING-targeted immunotherapy enhances natural killer cell- and CD8(+) T cell-mediated anti-tumor immunity. Biomaterials.

[12] Junkins R.D., Gallovic M.D., Johnson B.M., Collier M.A., Watkins-Schulz R., Cheng N., David C.N., McGee C.E., Sempowski G.D., Shterev I., McKinnon K., Bachelder E.M., Ainslie K.M., Ting J.P. (2018). A robust microparticle platform for a STING-targeted adjuvant that enhances both humoral and cellular immunity during vaccination. J. Control Release.

[13] Le Naour J., Zitvogel L., Galluzzi L., Vacchelli E., Kroemer G. (2020). Trial watch: STING agonists in cancer therapy. Oncoimmunology.

[14] Koshy S.T., Cheung A.S., Gu L., Graveline A.R., Mooney D.J. (2017). Liposomal delivery enhances immune activation by STING agonists for cancer immunotherapy. Adv. Biosyst..

[15] Nguyen T.A., Pang K.C., Masters S.L. (2017). Intercellular communication for innate immunity. Mol. Immunol..

[16] Kalluri R., LeBleu V.S. (2020). The biology, function, and biomedical applications of exosomes. Science.

[17] Pitt J.M., Kroemer G., Zitvogel L. (2016). Extracellular vesicles: Masters of intercellular communication and potential clinical interventions. J. Clin. Invest..

[18] Robbins P.D., Morelli A.E. (2014). Regulation of immune responses by extracellular vesicles. Nat. Rev. Immunol..

[19] Rutman A.K., Negi S., Gasparrini M., Hasilo C.P., Tchervenkov J., Paraskevas S. (2018). Immune response to extracellular vesicles from human islets of Langerhans in patients with type 1 diabetes. Endocrinology.

[20] Thery C., Ostrowski M., Segura E. (2009). Membrane vesicles as conveyors of immune responses. Nat. Rev. Immunol..

[21] Gutierrez-Vazquez C., Villarroya-Beltri C., Mittelbrunn M., Sanchez-Madrid F. (2013). Transfer of extracellular vesicles during immune cell-cell interactions. Immunol. Rev..

[22] Batrakova E.V., Kim M.S. (2015). Using exosomes, naturally-equipped nanocarriers, for drug delivery. J. Control Release.

[23] Kalluri R. (2016). The biology and function of exosomes in cancer. J. Clin. Invest..

[24] Luan X., Sansanaphongpricha K., Myers I., Chen H., Yuan H., Sun D. (2017). Engineering exosomes as refined biological nanoplatforms for drug delivery. Acta Pharmacol. Sin.

[25] Torralba D., Baixauli F., Villarroya-Beltri C., Fernandez-Delgado I., Latorre-Pellicer A., Acin-Perez R., Martin-Cofreces N.B., Jaso-Tamame A.L., Iborra S., Jorge I., Gonzalez-Aseguinolaza G., Garaude J., Vicente-Manzanares M., Enriquez J.A., Mittelbrunn M. (2018). Priming of dendritic cells by DNA-containing extracellular vesicles from activated T cells through antigen-driven contacts. Nat. Commun..

[26] Mendt M., Kamerkar S., Sugimoto H., McAndrews K.M., Wu C.C., Gagea M., Yang S., Blanko E.V.R., Peng Q., Ma X., Marszalek J.R., Maitra A., Yee C., Rezvani K., Shpall E. (2018). Generation and testing of clinical-grade exosomes for pancreatic cancer. JCI Insight.

[27] Kamerkar S., LeBleu V.S., Sugimoto H., Yang S., Ruivo C.F., Melo S.A., Lee J.J., Kalluri R. (2017). Exosomes facilitate therapeutic targeting of oncogenic KRAS in pancreatic cancer. Nature.

[28] Reus J.B., Trivino-Soto G.S., Wu L.I., Kokott K., Lim E.S. (2020). SV40 large T antigen is not responsible for the loss of STING in 293T cells but can inhibit cGAS-STING interferon induction. Viruses.

[29] Ma Z., Jacobs S.R., West J.A., Stopford C., Zhang Z., Davis Z., Barber G.N., Glaunsinger B.A., Dittmer D.P., Damania B. (2015). Modulation of the cGAS-STING DNA sensing pathway by gammaherpesviruses. Proc. Natl. Acad. Sci. U. S. A..

[30] Zhang Y., Yeruva L., Marinov A., Prantner D., Wyrick P.B., Lupashin V., Nagarajan U.M. (2014). The DNA sensor, cyclic GMP–AMP synthase, is essential for induction of IFN-β during *Chlamydia trachomatis* infection. J. Immunol..

[31] Luteijn R.D., Zaver S.A., Gowen B.G., Wyman S.K., Garelis N.E., Onia L., McWhirter S.M., Katibah G.E., Corn J.E., Woodward J.J., Raulet D.H. (2019). SLC19A1 transports immunoreactive cyclic dinucleotides. Nature.

[32] LeBleu V.S., Kalluri R. (2020). Exosomes as a multicomponent biomarker platform in cancer. Trends Cancer.

[33] Gentili M., Kowal J., Tkach M., Satoh T., Lahaye X., Conrad C., Boyron M., Lombard B., Durand S., Kroemer G., Loew D., Dalod M., Thery C., Manel N. (2015). Transmission of innate immune signaling by packaging of cGAMP in viral particles. Science.

[34] Diamond J.M., Vanpouille-Box C., Spada S., Rudqvist N.P., Chapman J.R., Ueberheide B.M., Pilones K.A., Sarfraz Y., Formenti S.C., Demaria S. (2018). Exosomes shuttle TREX1-sensitive IFN-stimulatory dsDNA from irradiated cancer cells to DCs. Cancer Immunol. Res..

[35] Baird J.R., Bell R.B., Troesch V., Friedman D., Bambina S., Kramer G., Blair T.C., Medler T., Wu Y., Sun Z., de Gruijl T.D., van de Ven R., Leidner R.S., Crittenden M.R., Gough M.J. (2018). Evaluation of explant responses to STING ligands: Personalized immunosurgical therapy for head and neck squamous cell carcinoma. Cancer Res..

[36] Vanpouille-Box C., Alard A., Aryankalayil M.J., Sarfraz Y., Diamond J.M., Schneider R.J., Inghirami G., Coleman C.N., Formenti S.C., Demaria S. (2017). DNA exonuclease Trex1 regulates radiotherapy-induced tumour immunogenicity. Nat. Commun..

[37] Zhou X., Kalluri R. (2020). Biology and therapeutic potential of mesenchymal stem cell-derived exosomes. Cancer Sci..

[38] Kugeratski F.G., Kalluri R. (2021). Exosomes as mediators of immune regulation and immunotherapy in cancer. FEBS J..

