# Supplementary material for: Effective delivery of STING agonist using exosomes suppresses tumor growth and enhances antitumor immunity
Source: J Biol Chem. 2021 Mar 9;296:100523. doi: 10.1016/j.jbc.2021.100523 (PMC8042450; doi:10.1016/j.jbc.2021.100523)
Supplement: Supplementary file 1 — Figures S1 to S3 [file mmc1.pdf]

# Supporting Information

# Effective delivery of STING agonist using exosomes suppresses tumor growth and enhances anti-tumor immunity

**Kathleen M. McAndrews[1], Sara P.Y. Che[1], Valerie S. LeBleu[1,2], Raghu Kalluri[1,3,4]**

[1]*Department of Cancer Biology, University of Texas MD Anderson Cancer Center, Houston, TX 77054, USA*

[2]*Feinberg School of Medicine, Northwestern University, Chicago, Illinois, USA*

[3]*Department of Bioengineering, Rice University, Houston, Texas, USA.*

[4]*Department of Molecular and Cellular Biology, Baylor College of Medicine, Houston, Texas, USA.*

## Supplementary Figure Legends

**Supplementary Figure 1**

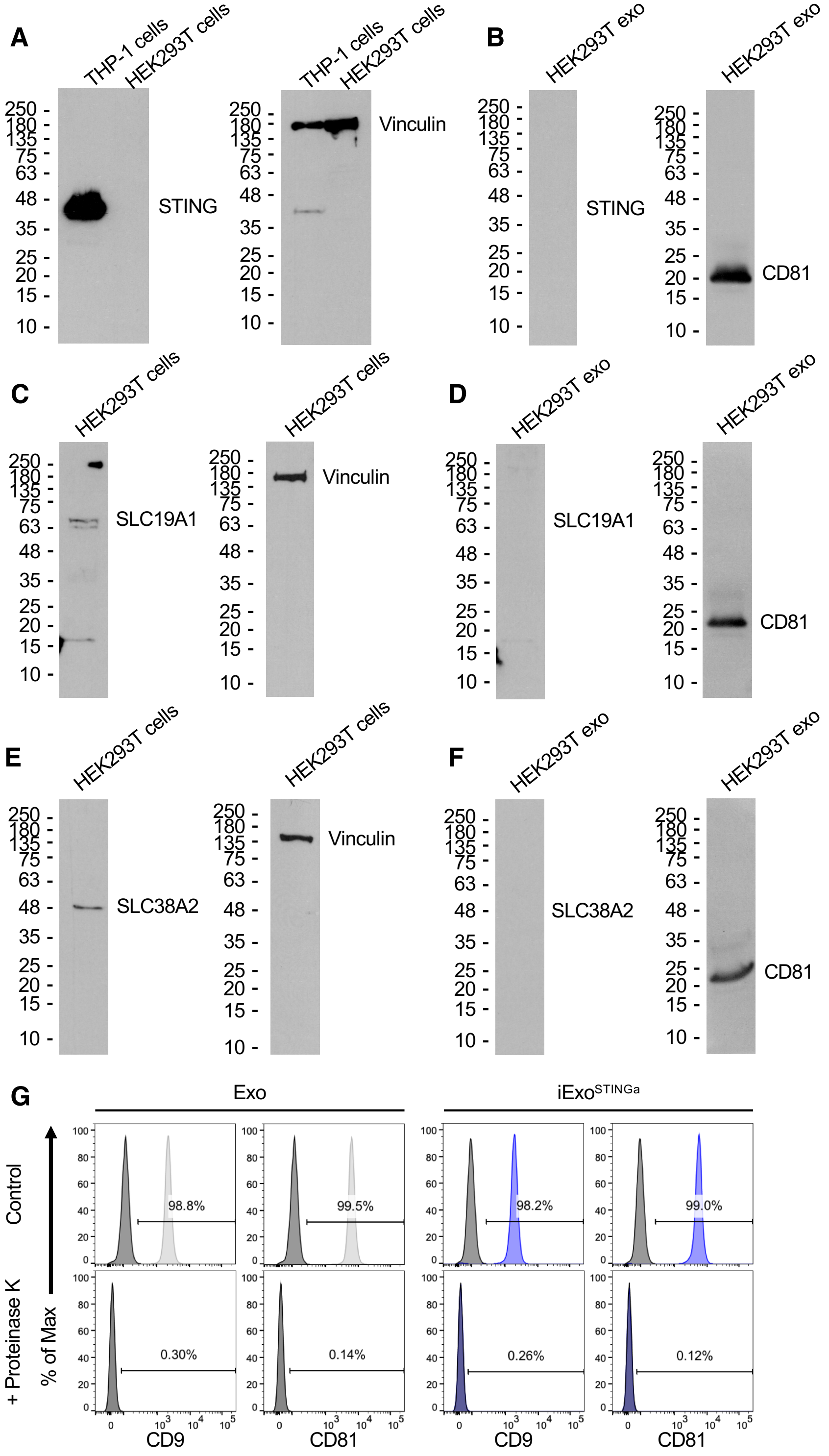

**Supplementary Figure 1. Protein levels of STING and transporters in exosomes**

**A.** Representative Western blot for STING in THP-1 and HEK293T cell lysates. Loading control, vinculin. **B**. Representative Western blot for STING in HEK293T exosomes (Exo). Loading control, CD81. **C**. Representative Western blot for SLC19A1 in HEK293T cell lysates. Loading control, vinculin. **D**. Representative Western blot for SLC19A1 in HEK293T exosomes. Loading control, CD81. **E**. Representative Western blot for SLC38A2 in HEK293T cell lysates. Loading control, vinculin. **F**. Representative Western blot for SLC38A2 in HEK293T exosomes. Loading control, CD81. **G**. Representative FACS histograms of CD9 and CD81 on mock treated (control) and Proteinase K treated HEK293T exosomes and $iExo^{STINGa}$.

## Supplementary Figure 2

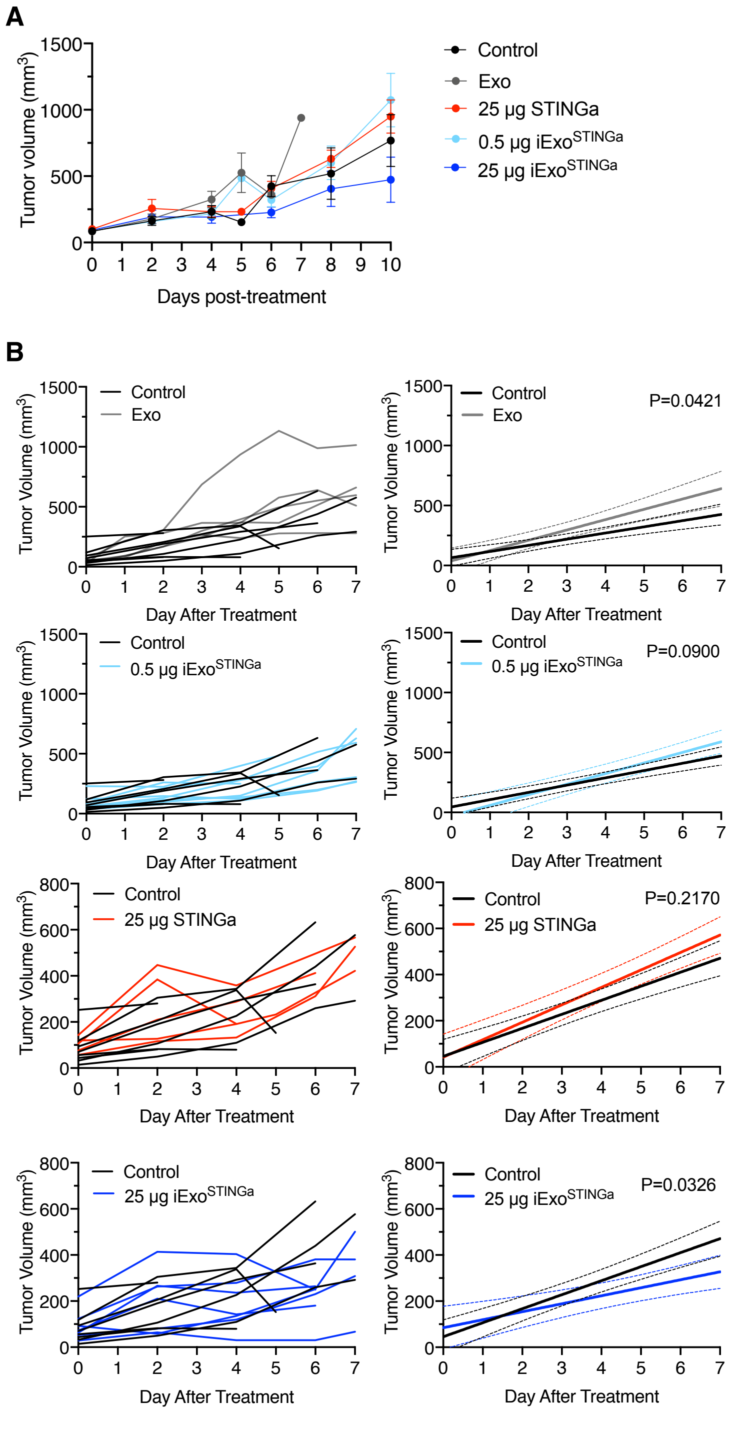


**Supplementary Figure 2. Anti-tumor activity of iExo$^{STINGa}$ loaded with 0.5 and 25 µg of STINGa**

**A.** Tumor volume over time in select groups. **B.** Tumor volume over time of individual mice in the indicated groups (left panel). Tumor volume over time in select groups and linear regression analysis testing for significant differences in slope (right panel). Panel (**B**) includes data also presented in Figure 2B: Control,

black lines; 25 μg STINGa, red lines; 25 μg $iExo^{STINGa}$, blue lines. The data is presented again in this panel to allow for direct comparison across all groups.

# Supplementary Figure 3

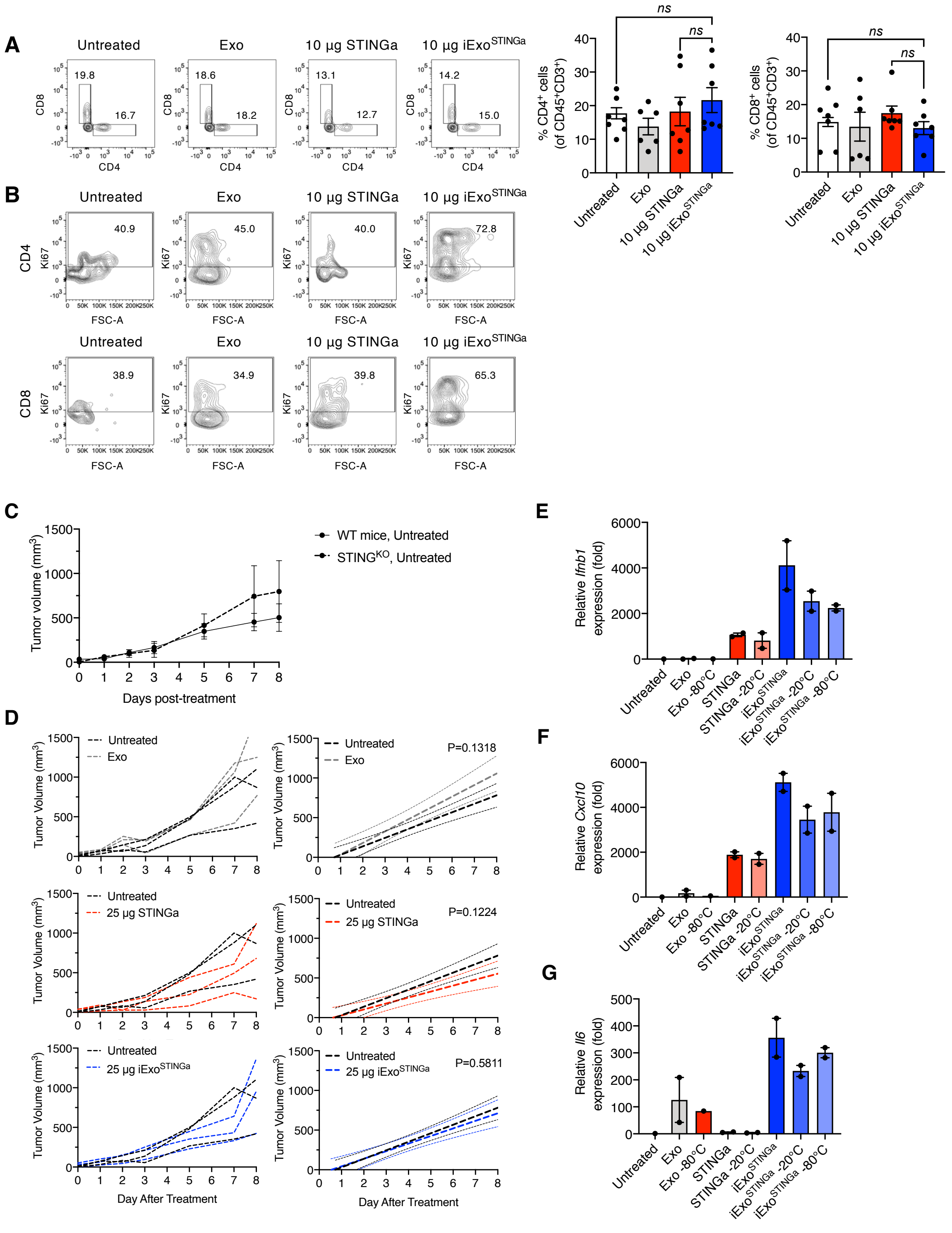


**Supplementary Figure 3. Analyses of ipsilateral and contralateral tumors in iExo$^{STINGa}$ treated mice, tumor growth kinetics in STING$^{KO}$ mice, and iExo$^{STINGa}$ stability**

**A**. Representative FACS plots and quantification of $CD4^+$ (left graph) and $CD8^+$ (right graph) cells in the tumors of the indicated groups, showing the ipsilateral tumor. Kruskall-Wallis with Dunn's multiple comparison test performed. **B**. Representative FACS plots for the quantification of $CD4^+Ki67^+$ (top panel) and $CD8^+Ki67^+$ (bottom panel) cells in the tumors of the indicated groups, showing the ipsilateral tumor in Figure 4E. The data are presented as the mean ± SEM. ns: not significant. **C**. Tumor volume over time in select groups. **D.** Tumor volume over time of individual $STING^{KO}$ mice in the indicated groups (left panel). Tumor volume over time in select groups and linear regression analysis testing for significant differences in slope (right panel). **E-G**. Relative gene expression (fold change relative to untreated cells) of *Ifnb1* (**E**), and *Cxcl10* (**F**), and *Il6* (**G**) in BMDCs treated with STINGa or $iExo^{STINGa}$ freshly prepared or stored at -20°C or -80°C. The data are presented as the mean ± SEM. Panel (**D**) includes data also presented in Figure 4G: Control, black lines; 25 µg $iExo^{STINGa}$, blue lines. The data is presented again in this panel to allow for direct comparison across all groups.
